# Blue space quality, quantity, and happiness in China: integrating subjective perceptions and objective indicators in a multilevel analysis

**DOI:** 10.3389/fpsyg.2025.1728308

**Published:** 2026-01-13

**Authors:** Fuxiang Yu, Chuntian Lu, Zhengbing Guo

**Affiliations:** 1 Business School, Hangzhou City University, Hangzhou, China; 2 School of Humanities and Social Science, Xi'an Jiaotong University, Xi‘an, China

**Keywords:** objective blue space, subjective blue space, perceived blue space quality, perceived blue space quantity, happiness

## Abstract

**Introduction:**

Human wellbeing is closely linked to interactions with natural environments, yet research on blue space has primarily focused on proximity to water bodies, paying limited attention to ecological quality, resource sufficiency, and the integration of subjective perceptions with objective conditions.

**Methods:**

This study examines how both subjective and objective dimensions of blue space influence individual happiness in China. We used data from the 2021 Chinese General Social Survey, linked with provincial-level ecological indicators from the China Statistical Yearbook. Multilevel ordered logistic regression models were applied to assess the associations between blue space indicators and happiness, with particular attention to cross-level interactions.

**Results:**

The results show that blue space quality and quantity contribute to happiness in distinct ways. At the individual level, more favorable perceptions of water quality and water sufficiency are associated with higher happiness. At the provincial level, higher wastewater treatment rates and greater groundwater supply are positively associated with happiness, whereas higher ammonia nitrogen emissions and excessive surface water resources are negatively associated. Cross-level analyses further indicate that the positive effect of perceived water quality on happiness weakens in provinces with higher levels of pollution.

**Discussion:**

These findings extend the Biophilia Hypothesis by demonstrating that, in addition to green spaces, contact with blue spaces enhances happiness through both subjective perceptions and objective environmental conditions. The results highlight the importance of sustainable water resource management for maximizing the social benefits of blue space.

## 1 Introduction

Human contact with natural environments has a broad range of health and wellbeing benefits, including reduced morbidity, improved mental health, and enhanced subjective life satisfaction (van den Bosch and Ode Sang, 2017; Li J. et al., 2024). The Biophilia Hypothesis provides a theoretical foundation for this connection, arguing that humans possess an innate tendency to affiliate with nature because of evolutionary processes that tied survival to natural surroundings (Wilson, 1984). From this perspective, exposure to natural settings is not only aesthetically rewarding but also fosters psychological restoration and emotional stability, which in turn support human flourishing (Liu et al., 2022). Research on the relationship between natural environments and happiness has primarily focused on urban green spaces, such as parks, trees, and other forms of vegetation. Evidence shows that contact with greenery can alleviate stress, promote attention restoration, and increase subjective happiness across diverse populations (Twohig-Bennett and Jones, 2018; Zhang Y. et al., 2024). Large-scale reviews have documented consistent associations between green space exposure and reduced risks of depression and anxiety (Kleinschroth et al., 2024).

More recently, scholars and policymakers have become increasingly interested in the potential of blue spaces to promote health and happiness (Raad et al., 2025; de Sousa Silva et al., 2025). Blue space is generally defined as natural or man-made environments dominated by surface water, including rivers, lakes, wetlands, coasts, canals, and reservoirs (Völker and Kistemann, 2015; Grellier et al., 2017). These environments not only provide ecological services such as water supply and climate regulation but also aesthetic enjoyment and opportunities for recreation, which may enhance happiness (White et al., 2020).

Emerging studies confirm that blue spaces can yield substantial psychological benefits. Proximity to or interaction with water environments has been associated with reduced stress, lower prevalence of depression, and greater self-reported life satisfaction (Britton et al., 2020). Meta-analyses further indicate that blue spaces may exert restorative effects comparable to or greater than those of green spaces, particularly in relation to mood enhancement and relaxation (Wang et al., 2025; Garrett et al., 2019). These findings suggest that water environments are an important but understudied dimension of the human–nature relationship.

Despite these advances, the literature on blue spaces remains relatively underdeveloped compared to green spaces, and several critical gaps remain. On the one hand, existing studies have focused disproportionately on proximity and accessibility, while paying insufficient attention to the quality and quantity of blue spaces. Most research examines whether living close to water bodies correlates with happiness, but fewer studies explore whether the actual environmental quality, such as water cleanliness or pollution, influences these outcomes. Water pollution could reduce the restorative potential of blue spaces by increasing perceptions of risk or disgust (Huang et al., 2020). Likewise, water quantity, measured through precipitation or groundwater supply (Feng et al., 2025), is rarely examined as a determinant of happiness, despite being central to human security and ecological sustainability (Wang et al., 2024). On the other hand, there is a methodological limitation related to measurement. Much of the research relies on objective exposure measures, such as GIS-based proximity to water bodies or regional hydrological data (Nutsford et al., 2016). While these indicators are valuable, they may not fully capture how individuals actually experience their environments. Recent studies in environmental psychology suggest that subjective perceptions of environmental quality and sufficiency are often stronger predictors of mental health than purely objective conditions (Helbich, 2018; Wu et al., 2020). Perceived water quality may directly influence emotional security and trust in the environment, shaping happiness beyond what objective indicators reveal (Li and Zhou, 2020; He et al., 2023).

Building on these limitations, this study addresses several key research questions. First, how do blue space quality and blue space quantity influence individual happiness? While most studies emphasize proximity to water environments, few systematically investigate the dual roles of water quality and resource availability, even though these are fundamental dimensions of ecological systems. Second, do subjective perceptions and objective indicators exert similar or divergent effects on happiness? Prior research suggests that perceptions of environmental quality often outweigh objective conditions in predicting mental health (Yuan et al., 2018). Yet the interplay between subjective experiences and objective measures of water resources remains insufficiently understood. Third, how do cross-level interactions between individual perceptions and provincial-level ecological indicators shape happiness outcomes? While some studies highlight the importance of considering multi-level dynamics in environment–health relationships, there is limited empirical evidence on whether positive perceptions can buffer against degraded environments or whether objective improvements amplify psychological benefits.

This study makes two contributions to the literature on environmental psychology and happiness. On the one hand, it extends the application of the Biophilia Hypothesis by demonstrating that water resources influence happiness not only through their presence and proximity but also through their quality and quantity. In doing so, the study enriches theoretical debates on how humans' innate affinity for nature operates across multiple ecological dimensions. On the other hand, the study integrates subjective perceptions with objective ecological indicators within a multilevel framework. By drawing on individual-level data from the Chinese General Social Survey (CGSS, 2021) together with provincial-level water resource indicators, this dual strategy responds to recent calls for combining self-reported experiences with ecological data to more accurately assess the linkages between environmental conditions and happiness.

## 2 Materials and methods

### 2.1 Conceptual framework

**Figure 1:**
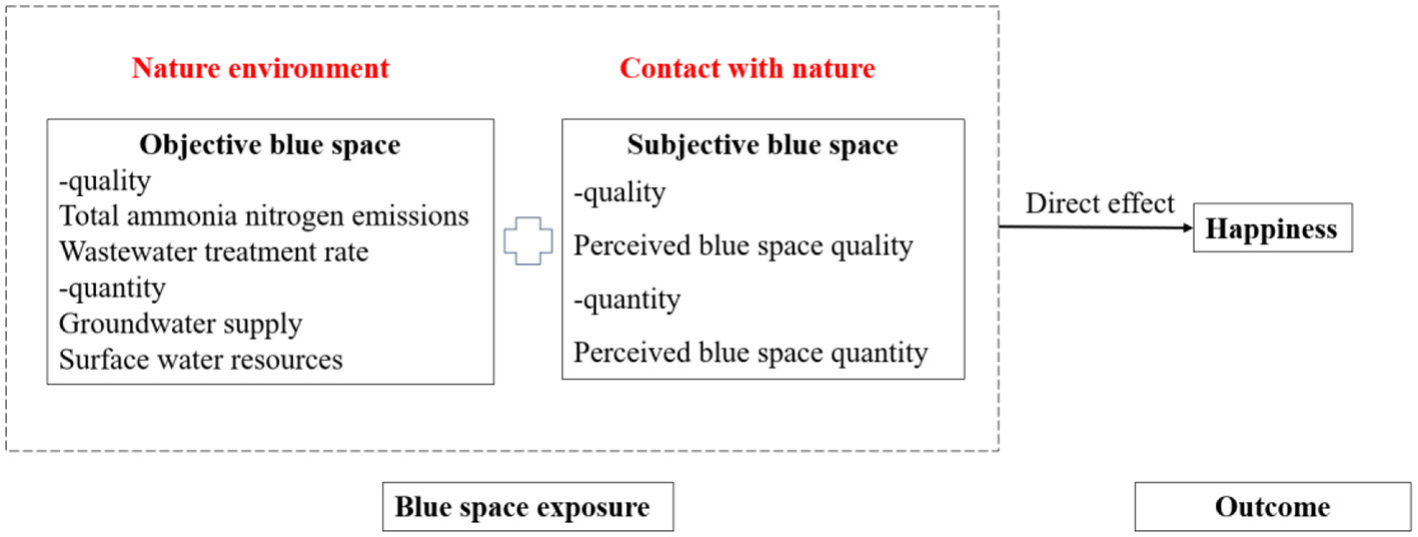
Research framework.

This study develops a conceptual framework in Figure 1 to examine how blue space influences individual happiness from both subjective and objective perspectives, grounded in the Biophilia Hypothesis. The Biophilia Hypothesis posits that humans possess an innate tendency to seek connections with nature, and that interaction with natural environments provides psychological and physiological benefits. Extending this perspective, we argue that blue space, as a vital component of the natural environment, contributes to human happiness not only through its presence but also through its ecological quality and sufficiency. Specifically, we distinguish between two fundamental dimensions of blue space: quality and quantity. At the individual level, subjective perceptions of water quality and water availability capture how residents evaluate their immediate environment and interpret potential risks or benefits. At the provincial level, objective measures of water pollution, wastewater treatment, surface water resources, and groundwater supply reflect the broader ecological context in which individuals are embedded. By integrating subjective perceptions and objective ecological conditions within a unified framework, this study provides a more comprehensive understanding of the pathways through which blue space influences happiness.

### 2.2 Data

This study employs data from the Chinese General Social Survey (CGSS) 2021, a nationally representative survey that provides rich individual- and household-level information across China. The CGSS, launched in 2003 and conducted biennially thereafter, is the earliest comprehensive social survey in China. It is designed and administered by the National Survey Research Center at Renmin University of China. The 2021 wave adopts a multi-stage stratified probability sampling method to ensure national representativeness. Specifically, the sampling process begins with the random selection of counties/districts, followed by the selection of communities/villages, households, and finally individual respondents aged 18 years or older. The CGSS 2021 dataset includes detailed information on demographics, socioeconomic status, health, and environmental perceptions, which makes it particularly suitable for examining the relationship between objective and subjective environmental conditions and individual happiness. For the purposes of this study, we restricted the sample to respondents with valid responses on key variables, including happiness, perceived blue space indicators, and relevant sociodemographic controls. After listwise deletion of missing values, the final analytic sample consists of 2,559 individuals nested within 19 provinces.

### 2.3 Measure

#### 2.3.1 Self-rated happiness

Self-rated happiness was measured following established research practices using the survey item “Overall, do you feel happy with your life?” (Diener et al., 2018). Respondents evaluated their general level of happiness on five response categories, ranging from “very unhappy” to “very happy.” Because only a very small proportion of participants reported being “very unhappy,” these responses were combined with “relatively unhappy” to create a single category representing unhappiness. Based on this adjustment, the variable was recoded into four ordered levels: unhappy (1), neutral (2), relatively happy (3), and very happy (4). This approach generates an ordinal measure of subjective happiness in which higher values reflect greater happiness. Such a recoding strategy enhances the robustness of the analysis.

#### 2.3.2 Perceived blue space

Perceived blue space in this study is conceptualized as comprising two distinct dimensions, namely perceived blue space quality and perceived blue space quantity. Perceived blue space quality was measured by asking respondents the question “In the past 12 months, to what extent has the community where you live been affected by water pollution?” The response categories were “a very large extent,” “a large extent,” “some extent,” “a little extent,” and “not at all.” Answers were coded on a five-point scale ranging from 1 (a very large extent) to 5 (not at all), with higher values indicating better perceived water quality. Perceived blue space quantity was assessed with the item “How serious is the problem of freshwater shortage in the area where you live?” Response options were “very serious,” “relatively serious,” “average,” “not very serious,” “not serious,” and “no such problem.” It is important to note that the option “no such problem” was explicitly distinguished from “unable to answer,” thereby reflecting the highest level of recognition regarding local water sufficiency rather than uncertainty or non-response. In this scale, higher values represent greater perceived water availability.

#### 2.3.3 Objective blue space

Similar to the subjective indicators, objective blue space was also divided into two dimensions, water quality and water quantity, with all indicators derived from the China Statistical Yearbook. Water quality was measured using two provincial-level indicators. The first indicator captured the emission aspect, measured by the total amount of ammonia nitrogen emissions in each province, which serves as a proxy for wastewater pollution levels. The second indicator reflected the treatment aspect, measured by the wastewater treatment rate of each province, which indicates the effectiveness of water quality management. Water quantity was assessed from two perspectives, resource ownership and supply. The ownership dimension was measured by the total volume of surface water resources in each province, reflecting the overall natural endowment of available water. The supply dimension was represented by the volume of groundwater supply, indicating the extent to which provinces rely on stable water sources to meet both ecological and human demands. To ensure comparability across provinces and facilitate regression analysis, all four indicators were standardized prior to inclusion in the models.

#### 2.3.4 Control variable

To account for potential confounding influences, a series of socioeconomic and demographic covariates were included in the models. Gender was coded as a binary variable, with 1 indicating male and 0 indicating female. Age was calculated by subtracting the respondent's year of birth from 2021. Marital status was coded as 1 for married respondents and 0 for all others. Political status distinguished members of the Communist Party of China, the Communist Youth League, or democratic parties (coded as 1) from the general population (coded as 0). Household economic status was measured on a five-point scale ranging from 1 (far below average) to 5 (far above average). Self-rated physical health was also measured on a five-point scale, with values from 1 (very unhealthy) to 5 (very healthy). Ethnicity was coded as 1 for ethnic minority respondents and 0 for Han Chinese. Education level was categorized into four groups, with 1 representing primary school or below, 2 representing junior high school, 3 representing senior high school, and 4 representing a bachelor's degree or above. Finally, the place of interview was coded with 1 representing resident committees and 0 representing village committees.

#### 2.3.5 Analytical strategy

To evaluate the relationship between blue space and individual happiness, we employed a multilevel ordinal logistic regression model with random intercepts. This modeling approach is appropriate because the dependent variable, self-rated happiness, is measured on an ordinal scale, and the data have a hierarchical structure with individuals nested within provinces. The analysis proceeded in several steps. First, we estimated a null model to calculate the intraclass correlation coefficient (ICC) and assess whether multilevel modeling was necessary. Second, we sequentially introduced individual level predictors, including subjective blue space indicators and control variables, as well as provincial level predictors, including objective blue space indicators, to examine their independent effects on happiness. Finally, we tested cross level interaction effects to explore whether the impact of subjective blue space perceptions on happiness varied depending on the objective environmental conditions of each province. All variance inflation factor (VIF) values were below 2.06, indicating no evidence of multicollinearity that would bias coefficient estimation.

## 3 Results

### 3.1 Descriptive characteristics

Table 1 presents the descriptive statistics for the variables included in the analysis. Among the 2,559 respondents, a majority reported being relatively happy (57.444 percent), followed by very happy (24.189 percent), neutral (12.778 percent), and unhappy (5.588 percent). This distribution suggests that most individuals in the sample perceive themselves as happy, with relatively few reporting low levels of happiness. For subjective measures of blue space, the mean score for perceived blue space quality was 4.166 (SD = 0.989) on a five-point scale, indicating that most respondents perceived relatively good water quality in their communities. Perceived blue space quantity had a mean of 4.453 (SD = 1.418) on a six-point scale, also suggesting a generally positive assessment of local water sufficiency. At the provincial level, the average ammonia nitrogen emission was 33,944.080 tons (SD = 17,465.700), while the average wastewater treatment rate was 96.752 percent (SD = 1.901). In terms of blue space quantity, the mean surface water resources amounted to 77.4028 billion cubic meters (SD = 50.7941 billion), while the average groundwater supply was 2.2798 billion cubic meters (SD = 2.8044 billion). These figures capture considerable heterogeneity in provincial water resources. Because the provincial indicators differ markedly in units and scales, all four provincial variables (surface water resources, groundwater supply, ammonia nitrogen emissions, and wastewater treatment rate) were standardized using Z-scores prior to model estimation. The standardized values were used in all multilevel regression models to facilitate coefficient comparability and improve numerical stability. Regarding control variables, the average age of respondents was 51.242 years (SD = 17.579), with the sample consisting of 53.615 percent females. About 30.637 percent of respondents were married, and 79.953 percent were classified as masses. Ethnic minorities accounted for 7.503 percent of the sample. The mean household economic status was 2.598 (SD = 0.788) on a five-point scale, and the mean self-rated health was 3.523 (SD = 1.101), indicating moderate variation in both economic and health conditions. In terms of educational attainment, the average was 2.281 (SD = 1.132), corresponding to an education level between junior and senior high school. Finally, 55.412 percent of interviews were conducted at community-level committees, while 44.588 percent took place in village-level committees.

**Table 1:** Descriptive statistics (*N* = 2,559).

**Variable**	**M or %**	**SD**	**Range**
**Happiness**			
Unhappy	5.588%		1
Neutral happy	12.778%		2
Relatively happy	57.444%		3
Very happy	24.189%		4
Perceived blue space quality	4.166	0.989	1–5
Perceived blue space quantity	4.45	1.42	1–6
Ammonia nitrogen emissions	33,944.080	17,465.700	2,192–57,519
Ammonia nitrogen emissions (Z-score)	0	1	−1.818–1.350
Wastewater treatment rate	96.752	1.901	92.720–99.540
Wastewater treatment rate (Z-score)	0	1	−2.121–1.466
Groundwater supply	22.789	28.044	0.200–96.900
Groundwater supply (Z-score)	0	1	−0.805–2.643
Surface water resources	774.028	507.941	7.500–1,783.600
Surface water resources (Z-score)	0	1	−1.509–2.988
Female	53.615%		0 = no, 1 = yes
Age	51.242	17.579	18–94
Married	30.637%		0 = no, 1 = yes
**Political status**			
Masses	79.953%		0 = no, 1 = yes
Communist Party/communist youth league/members of democratic parties	20.047%		0 = no, 1 = yes
Family economic status	2.598	0.788	1–5
Physical health	3.523	1.101	1–5
Han Chinese	92.497%		0 = no, 1 = yes
Education	2.281	1.132	1–4
**Place of interview**			
Resident committee	55.4%		0 = no, 1 = yes
Village committee	44.6%		0 = no, 1 = yes

### 3.2 The impact of blue space quality on happiness

We first assessed whether a multilevel modeling strategy was needed by estimating a null model with a provincial-level random intercept. The intraclass correlation coefficient (ICC) was 0.027, indicating that 2.7% of the variance in happiness was attributable to differences between provinces. Although the proportion appears modest, the clustering effect was statistically significant in the likelihood-ratio test, suggesting that ignoring provincial-level dependence may lead to biased standard errors. Based on this preliminary diagnostic, we then evaluated the necessity of multilevel modeling separately for the two analytical pathways. For the model examining the association between Blue Space Quality and happiness, the comparison between the single-level ordinal logistic model and the multilevel specification showed a significant improvement in model fit when provincial random intercepts were included (χ^2^ = 20.70, *p* < 0.001). This indicates that provincial-level variation plays a meaningful role in this pathway, and therefore a multilevel model is warranted. In contrast, for the model analyzing the relationship between Blue Space Quantity and happiness, the random intercept variance was nearly zero, and the likelihood-ratio test showed no improvement over the single-level specification (χ^2^ = 0.02, *p* = 0.44). These results suggest that provincial clustering is negligible in this case, making a single-level ordinal logistic model the more appropriate choice. Overall, the ICC results combined with the pathway-specific diagnostic tests indicate that multilevel modeling is appropriate for the Blue Space Quality analysis but unnecessary for the Blue Space Quantity analysis.

Table 2 reports the multilevel ordinal logistic regression results examining the relationship between blue space quality and self-rated happiness. Model 2 included only subjective blue space quality, which showed a positive and significant association with happiness (β = 0.087, *p* < 0.05). This suggests that individuals who perceived their local water quality more positively were more likely to report higher levels of happiness. In Model 3, we introduced the provincial-level indicator of ammonia nitrogen emissions. The results indicated a negative and significant effect (β = −0.180, *p* < 0.05), implying that higher levels of water pollution at the provincial level were associated with lower happiness. This finding highlights the detrimental impact of environmental degradation on individual happiness. Model 4 added wastewater treatment rate as another indicator of objective water quality. The coefficient was positive and significant (β = 0.196, *p* < 0.05), showing that higher levels of wastewater treatment were associated with greater happiness. This result underscores the importance of effective water governance in enhancing subjective happiness. Model 4 included both ammonia nitrogen emissions and wastewater treatment simultaneously. The positive effect of wastewater treatment remained significant (β = 0.158, *p* < 0.05), whereas the coefficient of ammonia nitrogen emissions became weaker and marginally significant (β = −0.132, *p* < 0.10). Finally, Model 6 examined the cross-level interaction between perceived blue space quality and total ammonia nitrogen emissions. The interaction term was negative and statistically significant (β = −0.100, *p* < 0.05), suggesting that the beneficial effect of perceiving higher blue space quality on happiness diminishes as objective pollution levels increase. In other words, while individuals who perceive their local water environment as clean and healthy tend to report higher levels of happiness, this positive association becomes weaker in provinces with higher emissions of ammonia nitrogen, a key indicator of water pollution.

**Table 2:** Multilevel ordered logistic regression results for blue space quality and happiness.

	**Model 1**	**Model 2**	**Model 3**	**Model 4**	**Model 5**	**Model 6**
Perceived blue space quality		0.087^*^ (0.041)	0.086^*^ (0.041)	0.089^*^ (0.041)	0.088^*^ (0.041)	0.097^*^ (0.043)
Ammonia nitrogen emissions			−0.180^*^ (0.083)		−0.132+ (0.078)	0.275 (0.168)
Wastewater treatment rate				0.196^*^ (0.080)	0.158^*^ (0.080)	0.134+ (0.077)
Total ammonia nitrogen emissions × Perceived blue space quality						−0.100^*^ (0.042)
Gender	−0.117 (0.080)	−0.120 (0.080)	−0.118 (0.080)	−0.119 (0.080)	−0.117 (0.080)	−0.125(0.080)
Age	0.021^***^ (0.003)	0.020^***^ (0.003)	0.020^***^ (0.003)	0.020^***^ (0.003)	0.020^***^ (0.003)	0.020^***^ (0.003)
Marital status	0.264^**^ (0.089)	0.263^**^ (0.089)	0.265^**^ (0.089)	0.267^**^ (0.089)	0.268^**^ (0.089)	0.269^**^ (0.089)
Political status	0.419^***^ (0.109)	0.419^***^ (0.109)	0.413^***^ (0.109)	0.421^***^ (0.109)	0.416^***^ (0.109)	0.414^***^(0.109)
Family economic	0.517^***^ (0.056)	0.517^***^ (0.056)	0.521^***^ (0.056)	0.515^***^ (0.056)	0.519^***^ (0.056)	0.522^***^(0.056)
Physical health	0.416^***^ (0.042)	0.413^***^ (0.042)	0.417^***^ (0.042)	0.412^***^ (0.042)	0.415^***^ (0.042)	0.416^***^ (0.042)
Ethnicity	0.049 (0.173)	0.053 (0.173)	0.049 (0.171)	0.006 (0.171)	0.013 (0.170)	−0.017 (0.169)
Education	0.065 (0.051)	0.065 (0.051)	0.062(0.051)	0.067 (0.051)	0.064 (0.051)	0.069 (0.051)
Place of interview	0.027 (0.091)	0.026 (0.091)	0.017 (0.091)	0.023 (0.090)	0.016 (0.090)	0.001 (0.090)
Cut1	1.107^***^ (0.308)	1.416^***^ (0.340)	1.437^***^ (0.339)	1.420^***^ (0.337)	1.435^***^ (0.336)	1.490^***^ (0.331)
Cut2	2.552^***^ (0.305)	2.863^***^ (0.339)	2.884^***^ (0.337)	2.867^***^ (0.336)	2.883^***^ (0.335)	2.940^***^ (0.330)
Cut3	5.503^***^ (0.324)	5.818^***^ (0.357)	5.842^***^ (0.355)	5.823^***^ (0.354)	5.840^***^ (0.353)	5.904^***^ (0.349)
Var(province)	0.109^*^ (0.050)	0.111^*^ (0.050)	0.090^*^ (0.041)	0.075^*^ (0.037)	0.067^*^ (0.033)	0.000 (0.000)
Var(perceived blue space quality)						0.004^*^ (0.002)
Sample size	2,559	2,559	2,559	2,559	2,559	2,559
Number of provinces	19	19	19	19	19	19
Log likelihood	−2,607.543	−2,605.292	−2,602.970	−2,602.644	−2,601.191	−2,597.903
AIC	5,241.086	5,238.584	5,235.939	5,235.288	5,234.382	5,231.805

+ *p* < 0.10, ^*^p < 0.05, ^**^p < 0.01, ^***^p < 0.001.

**Figure 2:**
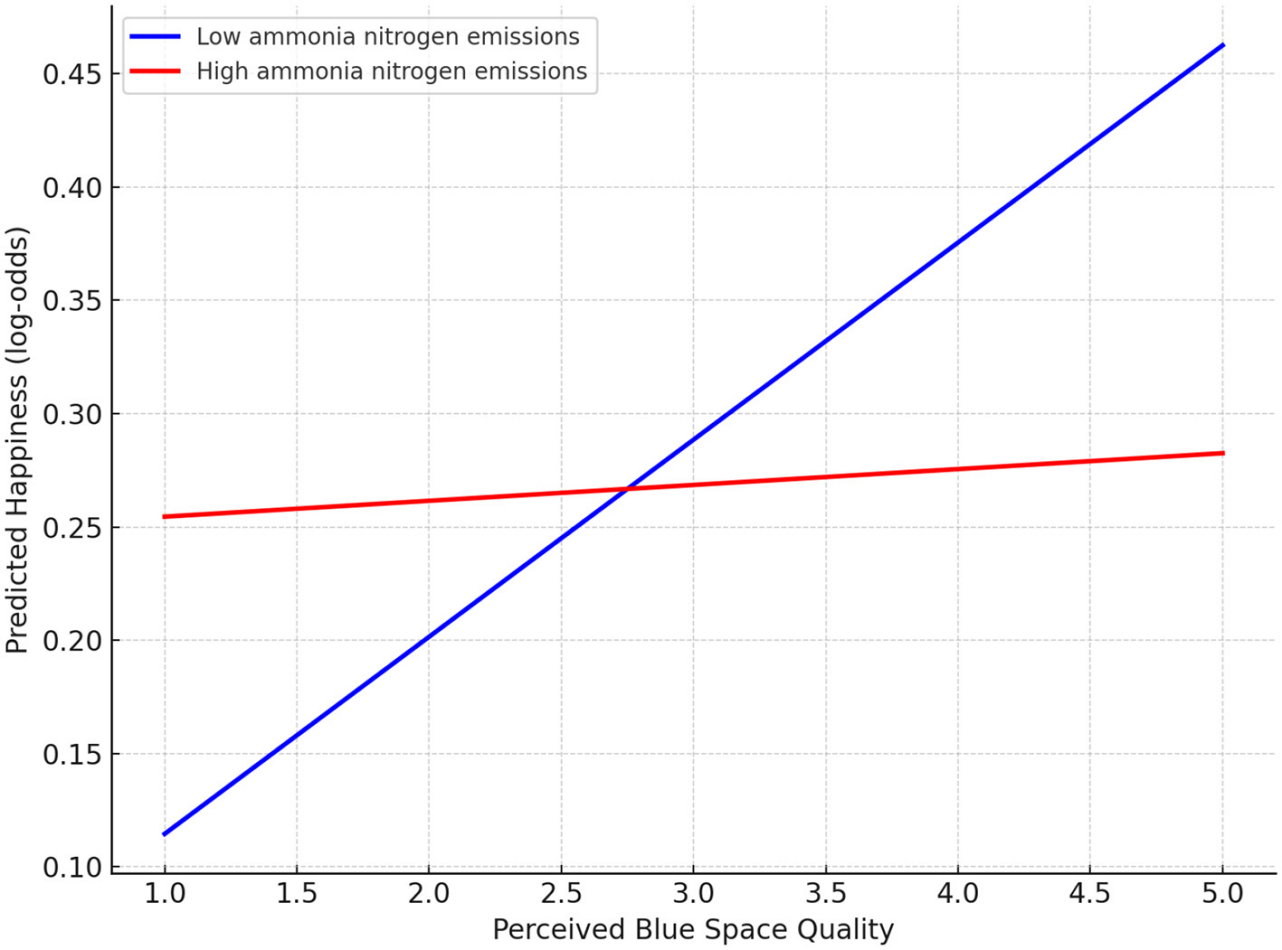
Moderating effect of ammonia nitrogen emissions on the relationship between perceived blue space quality and happiness.

To further clarify the cross-level interaction between perceived blue space quality and ammonia nitrogen emissions, Figure 2 depicts how the association between perceived quality and happiness changes under different levels of pollution. When ammonia nitrogen emissions are low, the slope is noticeably positive, indicating that higher perceived blue space quality is linked to greater happiness. The simple slope analysis confirms this pattern, showing that the effect of perceived quality is statistically significant under low-pollution conditions (*p* = 0.002). In contrast, when pollution levels are high, the slope becomes almost flat, suggesting that the influence of perceived quality tends to diminish or become ineffective in highly polluted environments. This interpretation is supported by the simple slope analysis, which shows that the effect of perceived quality is not statistically significant when ammonia nitrogen emissions are high (*p* = 0.657).

### 3.3 The impact of blue space quantity on happiness

Table 3 presents the results from the four ordered logistic regression models examining the association between blue space quantity and self-rated happiness. In Model 7, perceived blue space quantity shows a positive and marginally significant relationship with happiness (β = 0.052, *p* < 0.10). This finding suggests that individuals who perceive local water resources as more abundant tend to report higher levels of happiness. The effect becomes statistically significant in Model 8 (β = 0.066, *p* < 0.05) and remains consistently positive in Models 9 and 10, indicating a stable beneficial influence of perceived water sufficiency. Model 8 incorporates surface water resources as an objective provincial-level indicator. The coefficient is negative and highly significant (β = −0.220, *p* < 0.001). This pattern indicates that higher levels of surface water resources are associated with lower happiness. Although the direction may appear counterintuitive, it is possible that abundant surface water reflects challenges such as flooding risks, poor drainage, or inefficient water management, which may diminish daily living comfort despite overall water availability. Model 9 focuses on groundwater supply. The coefficient is positive and highly significant (β = 0.276, *p* < 0.001). Model 10 includes both groundwater supply and surface water resources. Groundwater supply remains positive and strongly significant (β = 0.226, *p* < 0.001), whereas the negative association between surface water resources and happiness persists but becomes slightly weaker (β = −0.130, *p* < 0.05).

**Table 3:** Ordered logistic regression results or blue space quantity and happiness.

	**Model 7**	**Model 8**	**Model 9**	**Model 10**
Perceived blue space quantity	0.052+ (0.028)	0.066^*^ (0.028)	0.056^*^ (0.028)	0.064^*^(0.029)
Surface water resources		−0.220^***^ (0.040)		−0.130^***^ (0.044)
Groundwater supply			0.276^***^ (0.040)	0.226^***^ (0.044)
Gender	−0.135+ (0.080)	−0.112 (0.080)	−0.129 (0.080)	−0.117 (0.080)
Age	0.0209^***^ (0.0029)	0.0193^***^ (0.0029)	0.0201^***^ (0.0029)	0.0194^***^ (0.0029)
Marital status	0.272^**^ (0.088)	0.256^**^ (0.088)	0.267^**^ (0.088)	0.258^**^ (0.088)
Political status	0.414^***^ (0.108)	0.406^***^ (0.108)	0.427^***^ (0.108)	0.420^***^ (0.108)
Family economic	0.498^***^ (0.055)	0.522^***^ (0.055)	0.507^***^ (0.055)	0.519^***^ (0.055)
Physical health	0.403^***^ (0.041)	0.411^***^ (0.041)	0.397^***^ (0.041)	0.403^***^ (0.042)
Ethnicity	−0.028 (0.150)	0.088 (0.151)	0.069 (0.151)	0.120 (0.152)
Education	0.083+ (0.049)	0.054 (0.049)	0.070 (0.049)	0.055 (0.049)
Place of interview	0.028 (0.087)	0.011 (0.087)	0.108 (0.088)	0.083 (0.088)
Cut1	1.374 (0.308)	1.358 (0.309)	1.344 (0.309)	1.340 (0.309)
Cut2	2.809 (0.306)	2.797 (0.307)	2.787 (0.307)	2.784 (0.307)
Cut3	5.710 (0.324)	5.726 (0.326)	5.732 (0.326)	5.738 (0.326)
Sample size	2,559	2,559	2,559	2,559
Log likelihood	−2,620.945	−2,605.715	−2,596.790	−2,592.378
Pseudo R^2^	0.057	0.062	0.066	0.067

+ *p* < 0.10, ^*^ *p* < 0.05, ^**^ *p* < 0.01, ^***^p < 0.001

### 3.4 Sensitivity analysis

To assess the robustness of the main findings regarding the relationship between blue space and individual happiness, a series of sensitivity analyses were conducted using alternative model specifications (Table 4). These complementary models further evaluated whether the estimated effects were influenced by provincial outliers and weighting choices. All provincial-level indicators were standardized prior to modeling to ensure comparability and interpretability across variables measured on different scales.

**Table 4:** Sensitivity analyses using alternative specifications.

**Variables**	**Model 11**	**Model 12**	**Model 13**
Control variables	YES	YES	YES
Perceived blue space quantity	0.061^*^ (0.030)	0.063+ (0.034)	
Surface water resources	−0.117^*^ (0.050)	−0.181^***^ (0.049)	
Groundwater supply	0.228^***^ (0.044)	0.211^***^ (0.050)	
Perceived blue space quality			0.041 (0.036)
Ammonia nitrogen emissions			0.167 (0.130)
Wastewater treatment rate			0.163 (0.093)
Total ammonia nitrogen emissions × Perceived blue space quality			−0.074^*^ (0.030)
Cut1	1.264 (0.319)	1.445 (0.355)	1.313 (0.369)
Cut2	2.731 (0.316)	2.967 (0.358)	2.840 (0.350)
Cut3	5.664 (0.336)	6.005 (0.384)	5.884 (0.392)
Var(province)			0.083 (0.022)
Sample size	2,403	2,559	2,559
Log likelihood	−2,436.897	−2,582.028	−2,590.550
Pseudo R^2^/AIC	0.066	0.072	5,215.100

+ *p* < 0.10, ^*^ *p* < 0.05, ^**^ *p* < 0.01, ^***^ *p* < 0.001

Given the substantial heterogeneity in provincial water endowments, Model 11 re-estimated the effects of blue space quantity on happiness after excluding provinces with extremely high levels of surface water resources. This approach evaluates whether the main result is driven by a small number of hydrologically unique provinces. The results remain consistent with the baseline multilevel model: groundwater supply continues to exhibit a positive association with happiness, while surface water resources retain a negative coefficient. Model 12 incorporated survey sampling weights to account for the multistage probability sampling design of the dataset. The probability weights were applied because they reflect the original sampling structure, whereas the post-stratification weights are not fully compatible with multilevel modeling and may introduce distortions when provincial-level variance components are estimated. After applying the appropriate weights, the positive association between groundwater supply and happiness remained statistically robust. Model 13 extends the weighted specification by evaluating the cross-level interaction between provincial ammonia nitrogen emissions and individuals' perceived water quality. The interaction term is negative and statistically significant, aligning with the main cross-level model presented earlier.

## 4 Discussion

### 4.1 Blue space quality and happiness

Our findings reveal that both subjective and objective dimensions of water quality are significantly associated with individual happiness. At the subjective level, respondents who perceived local water quality as better consistently reported higher levels of happiness, echoing recent evidence that perceptions of drinking-water safety and environmental quality shape health attitudes and happiness (Miller et al., 2024). The mechanism may operate through psychological restoration, whereby perceiving water bodies as clean and unpolluted provides a sense of safety, reduces environmental stressors, and enhances restorative experiences, consistent with contemporary syntheses of Stress Recovery Theory and Attention Restoration Theory in blue-space research (Li L. et al., 2024).

At the objective level, the results are more complex. Ammonia nitrogen emissions were negatively associated with happiness, which is consistent with recent Chinese evidence that water-pollution burdens are linked to worse health and lower happiness (Gunko et al., 2022; Lin et al., 2022). By contrast, wastewater treatment rate showed a positive effect, indicating that effective environmental governance can enhance individual happiness and related satisfaction. This may occur through two mechanisms. On the one hand, improving water quality reduces disease risks and supports healthier living conditions, a link reinforced by national reviews of progress in China's aquatic environmental management (Wang J. et al., 2024). On the other hand, visible improvements in wastewater management may increase public trust in government capacity and thereby enhance life satisfaction indirectly, as suggested by studies connecting governance performance, trust, and environmental satisfaction (Jia and Lin, 2025). These findings underscore that objective water quality is not only an ecological indicator but also a social signal of institutional performance (Qi et al., 2024).

The interaction results further deepen this picture. We found that the positive association between perceived water quality and happiness was weaker in provinces with higher levels of objective pollution, which accords with evidence that objective environmental hazards can mute the benefits of favorable perceptions for happiness (Gomm and Bernauer, 2023). This suggests that while subjective evaluations are important, they cannot fully compensate for the negative externalities of poor environmental conditions. Residents may attempt to maintain positive perceptions, but objective degradation imposes unavoidable health and livelihood risks that diminish happiness (Xiong et al., 2024). This interplay highlights a critical insight that subjective perceptions and objective realities are not interchangeable but mutually reinforcing dimensions of environmental experience, and that alignment between improved conditions and favorable perceptions yields the greatest happiness gains (White et al., 2013).

### 4.2 Blue space quantity and happiness

The results regarding blue space quantity reveal a more nuanced relationship with happiness compared to water quality. At the subjective level, individuals who perceived freshwater resources as sufficient reported significantly higher happiness. This finding is consistent with previous studies demonstrating that perceptions of environmental sufficiency and security enhance psychological stability and life satisfaction (Wu et al., 2022). The mechanism may operate through reducing environmental uncertainty. When people believe that water scarcity is not a pressing issue in their community, they are less likely to experience stress related to resource insecurity, thereby supporting greater happiness. Such findings resonate with the broader literature on environmental security as a determinant of human happiness (Jepson et al., 2017). In contrast, the provincial-level results show divergent effects. Groundwater supply was positively associated with happiness, suggesting that stable and reliable sources of water contribute to happiness. This result can be interpreted in both ecological and socioeconomic terms. Ecologically, groundwater represents a dependable resource that buffers against seasonal variability in precipitation (Taylor et al., 2013). Socioeconomically, abundant groundwater supports agricultural productivity and urban water demand, which in turn improves living standards and enhances life satisfaction. Hence, groundwater acts as a stabilizing factor that links ecological resilience with human happiness.

Surface water resources, however, exhibited a negative association with happiness. Although a greater endowment of surface water might intuitively be expected to promote wellbeing, excessive volumes can generate adverse consequences such as flooding, crop damage, infrastructure disruption, and heightened risks of waterborne diseases (Ren et al., 2025). Empirical evidence from China further shows that regions with abundant surface water are often exposed to considerable hydrological risks. For instance, ground-monitoring studies in Guangzhou reveal that high surface runoff contributes to recurrent urban flooding, producing substantial socioeconomic and public safety impacts (Zhang X. et al., 2024). Research in the Yellow River Basin similarly indicates that areas with greater river discharge and flood exposure experience higher vulnerability and economic losses during hydrological extremes, highlighting the costs associated with concentrated water resources (Hu et al., 2022). From a psychological perspective, residing in areas characterized by frequent flooding or inadequate water management may heighten perceived environmental risks and feelings of insecurity, which in turn undermine happiness. These findings collectively suggest that water resources cannot be interpreted solely in terms of their absolute abundance. Their influence on wellbeing depends on the stability, usability, and safety of the water environment as well as residents' perceptions of associated risks (Viljanen et al., 2025).

Comparing groundwater supply with surface water resources shows that stable water stocks and excessive water abundance have very different implications for happiness. While stable and accessible sources such as groundwater enhance happiness by providing security and predictability, large volumes of surface water may act as an environmental stressor that undermines happiness despite increasing overall availability. This distinction advances the literature by demonstrating that “more water” does not automatically translate into “greater happiness.” Instead, the context, stability, and usability of water fundamentally shape its social consequences (Gray and Sadoff, 2007).

### 4.3 Practical implications

The findings of this study provide important implications for environmental governance and policy-making. First, the negative association between ammonia nitrogen emissions and happiness highlights the urgent need for stricter regulation of industrial wastewater and more effective pollution control strategies. Strengthening environmental enforcement not only improves ecological quality but also contributes directly to public happiness (Wang and Yang, 2016). Second, the positive role of wastewater treatment rates suggests that investments in water management infrastructure are critical for improving life satisfaction. Expanding wastewater treatment facilities and improving technological efficiency can simultaneously reduce environmental risks and enhance public trust in governance, a finding consistent with recent studies linking environmental governance capacity to improved quality of life (Wang C. et al., 2024). Third, the contrasting effects of groundwater supply and surface water resources indicate that water management strategies should not only emphasize abundance but also prioritize stability and risk mitigation. Ensuring the sustainable use of groundwater resources can provide a reliable buffer against seasonal variability and climate change impacts (Amanambu et al., 2020). At the same time, policies must address the risks associated with excessive or poorly managed surface water through improved allocation mechanisms, ecological restoration, and climate adaptation planning, since large volumes of surface water may undermine both the perceived and actual security of local environments (Liu et al., 2024). Finally, the importance of subjective perceptions underscores the role of public engagement and environmental communication. Policies that only improve objective conditions may not translate into higher happiness if residents remain unaware of or skeptical about these improvements. Enhancing transparency, promoting environmental education, and fostering citizen participation in water governance can strengthen positive perceptions, thereby maximizing the social benefits of blue space improvements (Liu et al., 2023).

### 4.4 Limitations and future research

Despite its contributions, this study has several limitations that should be acknowledged. First, the analysis relies on cross-sectional data from the 2021 wave of the CGSS, which restricts the ability to draw causal inferences between blue space and happiness. While multilevel modeling reduces bias from unobserved heterogeneity, longitudinal data would allow for stronger causal claims and the identification of dynamic processes over time. Future research should therefore employ panel data or natural experiments to better capture causal pathways.

Second, the measurement of key constructs such as self-rated happiness and perceived blue space relies on single-item indicators. Although single-item measures are widely used in large-scale surveys, they may be subject to measurement error and limited construct validity. Future studies should incorporate multi-item validated scales to provide a more robust assessment of subjective happiness and environmental perceptions.

Third, the objective indicators of blue space in this study were restricted to four provincial-level measures, namely ammonia nitrogen emissions, wastewater treatment rate, groundwater supply, and surface water resources. While these variables capture important aspects of water quality and quantity, they do not fully reflect the ecological complexity of blue space, such as ecosystem services, biodiversity, or access to urban water bodies. Incorporating a broader range of ecological and spatial indicators, potentially derived from remote sensing or ecological monitoring systems, would provide a more comprehensive assessment.

Finally, the mechanisms linking blue space to happiness remain only partially understood. While our study highlights the importance of both subjective perceptions and objective conditions, future research should explicitly test mediating pathways such as psychological restoration, social cohesion, and trust in government. Employing mixed-method approaches that combine survey data with qualitative interviews or experimental designs could deepen understanding of how blue spaces contribute to happiness (Vegaraju and Amiri, 2024).

## 5 Conclusion

This study set out to investigate how both subjective and objective dimensions of blue space influence individual happiness in China, addressing three central questions: (1) how blue space quality and quantity shape happiness, (2) whether subjective perceptions and objective indicators exert similar or divergent effects, and (3) how cross-level interactions between perceptions and ecological conditions affect happiness.

The findings provide clear answers to these questions. First, both blue space quality and quantity play important but distinct roles in shaping happiness. At the individual level, favorable perceptions of water quality and sufficiency are consistently associated with higher self-rated happiness. At the provincial level, better wastewater treatment and stronger groundwater supply enhance happiness, while higher ammonia nitrogen emissions and excessive surface water resources reduce it. These results confirm that happiness is determined not only by the abundance of water resources but also by their ecological integrity, stability, and usability. Second, subjective perceptions and objective indicators exert both complementary and contrasting effects. Perceived blue space quality and sufficiency serve as powerful psychological pathways that directly increase happiness, but their benefits are conditional on objective environmental realities. In provinces with high levels of water pollution, the positive effect of perceived water quality is significantly weakened, underscoring the need to integrate both subjective and objective perspectives in environmental psychology. Third, the multilevel analysis reveals important cross-level dynamics. Subjective perceptions can buffer against adverse conditions to some extent, but they cannot fully compensate for the detrimental effects of ecological degradation. Likewise, improvements in objective conditions amplify the benefits of positive perceptions, highlighting the interactive nature of environmental influences on happiness.

## Data Availability

Publicly available datasets were analyzed in this study. This data can be found at: http://cgss.ruc.edu.cn/.
